# Supplementary material for: End Sequence Analysis Toolkit (ESAT) expands the extractable information from single-cell RNA-seq data
Source: Genome Res. 2016 Oct;26(10):1397–410. doi: 10.1101/gr.207902.116 (PMC5052061; doi:10.1101/gr.207902.116)
Supplement: Supplemental Material [file supp_gr.207902.116_Supp_Materials.tar.gz › ESAT-esat_v0.1/doc/html/classbroad_1_1core_1_1sequence_1_1_fasta_quality_index.html]

ESAT: broad.core.sequence.FastaQualityIndex Class Reference


ESAT


- Main Page
- Related Pages
- Packages
- Classes
- Files

- Class List
- Class Index
- Class Hierarchy
- Class Members

All Classes Namespaces Files Functions Variables Enumerator Pages

- broad
- core
- sequence
- FastaQualityIndex

Classes |
Public Member Functions |
Static Public Member Functions |
List of all members

broad.core.sequence.FastaQualityIndex Class Reference

|  |  |
| --- | --- |
| Classes | |
| class | **SequenceFileLayout** |
|  | |

|  |  |
| --- | --- |
| Public Member Functions | |
|  | FastaQualityIndex () |
|  | |
| void | createIndexFromQualityFile (String fastaQualityFile, final long period) throws IOException |
|  | |
| void | loadFromAscii (String indexFile) throws IOException |
|  | |
| void | writeAsText (String outFile) throws IOException |
|  | |

|  |  |
| --- | --- |
| Static Public Member Functions | |
| static void | main (String[] args) throws Exception |
|  | |

## Constructor & Destructor Documentation

|  |  |  |  |  |
| --- | --- | --- | --- | --- |
| broad.core.sequence.FastaQualityIndex.FastaQualityIndex | ( |  | ) |  |

Here is the caller graph for this function:

## Member Function Documentation

|  |  |  |  |
| --- | --- | --- | --- |
| void broad.core.sequence.FastaQualityIndex.createIndexFromQualityFile | ( | String | *fastaQualityFile*, |
|  |  | final long | *period* |
|  | ) |  | throws IOException |

|  |  |  |  |  |  |
| --- | --- | --- | --- | --- | --- |
| void broad.core.sequence.FastaQualityIndex.loadFromAscii | ( | String | *indexFile* | ) | throws IOException |

|  |  |  |  |  |  |
| --- | --- | --- | --- | --- | --- |
| static void broad.core.sequence.FastaQualityIndex.main | ( | String[] | *args* | ) | throws Exception |

static

Here is the call graph for this function:

|  |  |  |  |  |  |
| --- | --- | --- | --- | --- | --- |
| void broad.core.sequence.FastaQualityIndex.writeAsText | ( | String | *outFile* | ) | throws IOException |

---

The documentation for this class was generated from the following file:

- java/broad/core/sequence/FastaQualityIndex.java


---

Generated on Fri Aug 15 2014 15:10:55 for ESAT by  

 1.8.7
